# GATA2 deficiency detected by newborn screening for SCID: A case report

**DOI:** 10.3389/fped.2022.1031106

**Published:** 2023-01-16

**Authors:** Alejandra Escobar Vasco, Larisa Broglie, Julie-An Talano, John Routes, James Verbsky, Allison Remiker

**Affiliations:** ^1^ Division of Hematology/Oncology/Blood and Marrow Transplantation, Department of Pediatrics, Medical College of Wisconsin and Children's Wisconsin, Milwaukee, WI, United States; ^2^ Division of Allergy and Clinical Immunology, Department of Pediatrics, Medical College of Wisconsin and Children's Wisconsin, Milwaukee, WI, United States; ^3^ Division of Rheumatology, Department of Pediatrics, Medical College of Wisconsin and Children's Wisconsin, Milwaukee, WI, United States

**Keywords:** GATA2, T cell receptor excision circles, severe combined immunodeficiency, hematopoietic stem cell transplant, inborn errors of immunity

## Abstract

The early diagnosis and treatment of inborn errors of immunity (IEI) is crucial in reducing the morbidity and mortality due to these disorders. The institution of newborn screening (NBS) for the diagnosis of Severe Combined Immune Deficiency (SCID) has decreased the mortality of this disorder and led to the discovery of novel genetic defects that cause this disease. GATA2 deficiency is an autosomal dominant, pleiotropic disease with clinical manifestations that include bone marrow failure, monocyte and B cell deficiency, leukemia, pulmonary alveolar proteinosis and lymphedema. We present the case of an infant identified by newborn screening for SCID due to GATA2 deficiency.

## Introduction

Severe combined immune deficiency (SCID) is a primary immune deficiency characterized by a profound deficiency in T cells and variable deficiencies in B and NK cells that is fatal if not detected and treated early in life (1). To date, more than 20 different genetic defects have been identified, thought to comprise approximately 90% of the causative defects in SCID (2). Early diagnosis and treatment of SCID before the acquisition of infections improves survival. Newborn screening for SCID using the T cell receptor excision circles (TRECs) assay has been implemented throughout the United States and several other countries. TRECs are a validated biomarker for newly formed naive T cells, which are decreased in all genetic causes of SCID and other causes of T cell lymphopenia. In numerous studies, the TREC assay has been shown to be a very sensitive assay to screen for SCID (3).

GATA2 deficiency syndrome has a wide phenotype including immunodeficiency, cytopenias, bone marrow failure and leukemia. The GATA family consist of 6 transcriptional factors that regulate gene expression by binding to the DNA motif GATA and other transcription factors *via* two zinc finger domains. Of the 6 GATA proteins, GATA1 and GATA2 play critical roles in hematopoiesis with GATA2 being a key transcriptional regulator required for the development and maintenance of a healthy stem cell pool (4, 5). Hematological manifestations of GATA2 deficiency include a range of peripheral cytopenias of which the most common are profound cytopenias of B-cells, NK cells, and monocytes, that tend to be progressive with loss of bone marrow progenitor populations over time and a tendency toward clonal hematopoiesis (6–8).

Evaluation of families with symptomatic relatives, have shown that the development of cytopenias is progressive and that there is partial penetrance with the identification of phenotypically normal patients amongst these family cohorts (7). The age of clinical onset is usually during childhood or early adolescence, and the penetrance is estimated at 90% by the age of 60 years (7). Herein, we describe a newborn infant presenting with undetectable TREC in her newborn screen that was found to have severe persistent T cell lymphopenia during the first 6 months of age secondary to GATA2 deficiency.

## Case description

An 8-day old female is referred to immunology clinic with an abnormal newborn screen reporting undetectable TRECs. Complete blood count (CBC) reported red blood cell macrocytosis for age, thrombocytopenia, and severe lymphopenia (Table 1). Lymphocyte immunophenotyping by flow cytometry showed markedly reduced naïve T cells, low/absent B cells and decreased NK cells (Absent bright CD56 cells) consistent with SCID phenotype (Table 2). She was started on Ig replacement therapy (SCIG), antimicrobial prophylaxis with acyclovir, fluconazole, and Trimethoprim-Sulfamethoxazole at 1-month of age. Thrombocytopenia resolved by 2-weeks of age.

**Table 1:** Patient's complete blood counts with age specific normal values are given on parenthesis. Trend over time.

	7 days old	15 days old	1 month old	3 months old	4 months old	5 months old	6 months old
RBC (10*6/µl)	4.41 (4.1–6.7)	3.99 (4.1–6.7)	3.69 (3.8–5.4)	4.33 (3.8–5.4)	4.26 (3.8–5.4)	4.7 (3.8–5.4)	4.89 (3.8–5.4)
Hemoglobin (g/dl)	18.3 (15–24)	16 (15–24)	13.3 (10.5–14)	13.4 (10.5–14)	12.5 (10.5–14)	13.1 (10.5–14)	12.9 (10.5–14)
MCV (fl)	134 (99–115)	126 (99–115)	108 (72–88)	90 (72–88)	84 (72–88)	82 (72–88)	79 (72–88)
Platelets (10*3/µl)	84 (150–450)	150 (150–450)	610 (150–450)	608 (150–450)	572 (150–450)	540 (150–450)	562 (150–450)
WBC (10*3/µl)	5.6 (5–21)	5.0 (5–21)	3.4 (6–14)	2.0 (6–14)	4.1 (6–14)	3.6 (6–14)	5.2 (6–14)
Absolute neutrophils	3.2 (1.4–13.4)	2.1 (1.4–13.4)	1.8 (1.3–6.7)	1.3 (1.3–6.7)	3.2 (1.3–6.7)	2.8 (1.3–6.7)	4.4 (1.3–6.7)
Absolute lymphocytes	0.2 (1.8–9.7)	0.7 (1.8–9.7)	0.6 (2.5–9.9)	0.4 (2.5–9.9)	0.3 (2.5–9.9)	0.4 (2.5–9.9)	0.1 (2.5–9.9)
Absolute Monocytes	1.7 (0.7–1.9)	1.9 (0.7–1.9)	0.8 (0.7–1.9)	0.3 (0.7–1.9)	0.4 (0.7–1.9)	0.3 (0.7–1.9)	0.4 (0.7–1.9)

**Table 2:** Patient's lymphocyte immunophenotyping given in absolute counts. Trend over time.

Age	ALC/mm^3^	CD3/mm^3^	CD4/mm^3^	CD8/mm^3^	CD19/mm^3^	CD56/mm^3^	CD45RA%	CD45RO%
7 days old	224	105	52	24	2	35	54	24
15 days old	450	270	90	171	0	31	6	0
1 months old	575	334	167	167	6	207	4	2
3 months old	380	304	171	137	4	57	64	0
4 months old	164	121	67	52	2	10	58.8	1.97
5 months old	180	148	81	67	2	18	52	1.01
6 months old	208	144	89	54	2	21	60.55	3.79

Her initial work up included molecular testing for SCID and T cell disorders (*ADA, CD3, CD45, DCLREIC, FOXN1, IL2RG, IL7R, JAK3, LIG4, NHEJ1, ORA1, RAG1/2, RMRP, STAT5B, STIM1, TBX1, and ZAP70*), telomere length measurement, and flow cytometry-based mitogen testing which were all normal. A limited exome that includes >4,800 genes (Trusight, Illumina) was performed and demonstrated a known heterozygous pathogenic variant in GATA2 (p.Thr354Met, c.1061C > T), which was confirmed by Sanger sequencing. Her pattern of lymphopenia of B cells and NK cells, followed by progressive monocytopenia for age, was consistent with GATA2 deficiency. Parental evaluation demonstrated that the mother harbored the same mutation, and even though, mother has not presented with any clinical manifestations of disease, she was noted to have 1.7% CD56 bright cells which is below published normal (9).

Complications during the first 8 months of life included medication induced elevated transaminases requiring discontinuation of fluconazole prophylaxis, chronic diarrhea with a negative infectious work up that self-resolved after 1 month, and an uncomplicated *Klebsiella oxytoca* urinary tract infection. Bone marrow aspirate and biopsy at 7 months of age revealed a mildly hypocellular marrow for age (80% cellularity) with no dysplasia, no excess blasts, negative FISH panel for MDS and normal cytogenetics. Serial monitoring of lymphocyte immunophenotyping continued to show severe T cell, B cell and NK cell cytopenias that fitted a phenotypic picture of severe combined immunodeficiency (Table 2). Since cytopenias did not recover and worsen over time (specifically T cells), the decision was made to proceed with hematopoietic stem cell transplant (HSCT) before she developed life threatening infections.

At 8 months of age, she received a matched unrelated donor bone marrow transplant (male, 34 y old, 10/10 HLA matched, CMV matched) following myeloablative conditioning with busulfan, fludarabine and equine anti-thymocyte globulin (ATG). She engrafted with 100% donor chimerism. She was able to be weaned off SCIG 7 months post-transplant and started vaccinations 2 months after SCIG was discontinued with evidence of seroconversion for vaccine induced antibodies for pneumococcus and *Haemophilus influenzae* type B. Immunosuppression was discontinued at 10 months post-transplant, and she was off prophylactic antimicrobials after 1 year.

**Figure 1:**
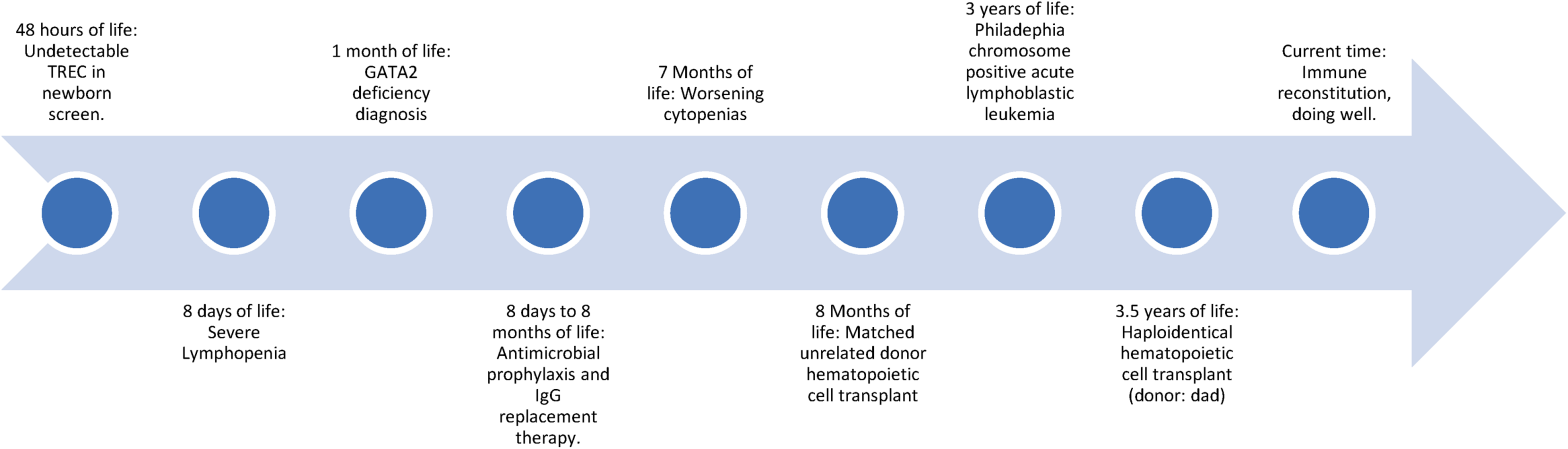
Timeline of events of patient described.

Two and a half years after transplant she was diagnosed with Philadelphia chromosome-positive acute lymphoblastic leukemia (Ph + ALL) that was found to be donor derived. She was treated with 4 drug induction chemotherapy per COG protocol AALL1131 and imatinib followed by consolidation with blinatumomab. She achieved complete remission. Two years and 9 months after her initial transplant, she received a second allogeneic HSCT, this time a haploidentical transplant using her father as a donor. The myeloablative conditioning regimen consisted of total body irradiation, thiotepa, cyclophosphamide, and ATG, with *αβ* T-cell and CD19 cell depletion as graft vs. host disease (GVHD) prophylaxis. She engrafted with 100% donor chimerism. She has mild, chronic skin GVHD but is currently 13 months post-second transplant and doing well (Figure 1). The patient's donor derived leukemia case has been reported elsewhere (10).

## Discussion

Since the recognition of GATA2 deficiency as the underlying cause of several clinical entities formerly known as MonoMAC syndrome, familial myelodysplastic syndrome (MDS)/acute myeloid leukemia (AML), DCML deficiency (dendritic cell, monocyte, B, and NK lymphoid deficiency), and Emberger syndrome, a lot has been learned about this genetic defect, and currently it is recognized as a frequent genetic cause for bone marrow failure and immune disorders in children and young adults. Deletions, mutations in regulatory regions, frameshift mutations, and substitutions have been described within the GATA2 locus without clear evidence of genotype-phenotype correlation. The clinical phenotype is highly variable and include asymptomatic carriers to early development of clinically significant cytopenias that evolve to MDS. The lack of genotype-phenotype correlation is highlighted in our patient and her mother, both with the same mutation and significantly different phenotypes, with her mother being asymptomatic to date. However, based on population studies that have reported unaffected individuals carrying GATA2 mutation into their fifth and sixth decades of life with a lifetime risk of MDS of approximately 90%, close monitoring of her mother is warranted (5, 7, 11–13).

The evolution of cytopenias in GATA2 deficiency syndrome has been described using symptomatic patients with DCML deficiency, and even though T cell cytopenias have been described, the profound and persistent T cell lymphopenia from birth in our patient is unusual and to our knowledge this early finding has not been described in GATA2 deficiency before (3, 7, 8). It is unclear if this presentation is simply a variable presentation of GATA2 deficiency, or if there are other genetic modifiers that may have affected these results. There were no clear defects that affect T cell development in the sequencing panel, but this possibility cannot be ruled out.

Early identification of children with inborn error of immunity (IEI) and its molecular constitution has paved the way for pre-symptomatic treatment and improved quality of life. With this goal in mind the TRECs assay was added to the NBS since 2008 and implemented in all 50 states by 2018 (3). The TRECs assay detects not only SCID but any condition causing low naïve T-cell counts, as described on our patient. Table 3 summarizes IEI other than SCID that have been diagnosed using NBS (3, 14). Several genetic panels are commercially available for the diagnosis of IEI that can usually apply to patients with certain clinical phenotypes or abnormal NBS. With a growing number of identifiable genetic mutations associated with specific phenotypes, panels may miss previously unidentified genes associated with disease. Next generation sequencing has become more affordable and available for clinical practice over the last several years, and whole-exome sequencing and whole-genome sequencing are becoming important diagnostic tools for identification of IEI. The ability to screen for genetic variants in many genes is important, as a variety of clinical and immunological phenotypes may result from mutations in a single gene (genetic pleiotropy), or mutations in multiple genes can underlie the same phenotype (genetic heterogeneity). In the case of our patient, initial negative molecular testing for SCID led to next generation sequencing and detection of the GATA2 variant. It was decided to use this diagnostic method to confirm the diagnosis, which proved to be a cost-effective approach for our group (15, 16).

**Table 3:** Causes of low TREC detected on newborn screen.

**Severe combined immunodeficiency**
• Typical • Leaky (Including Omenn Syndrome)
**Syndromes**
• DiGeorge • Trisomy 21 • Ataxia-Telangiectasia • CHARGE • Jacobsen syndrome • Barth syndrome • GATA2 deficiency
**Secondary**
• Congenital heart disease • Congenital gastrointestinal malformations: gastroschisis, intestinal atresia, meconium ileus • Hydrops

Table adapted from Currier R, Puck JM. SCID newborn screening: What we’ve learned. *J Allergy Clin Immunol*. 2021 Feb;147(2):417–426.

The genetic diagnosis of our patient allowed the identification of carrier status of her mother. Even though she continues to be asymptomatic to date, close monitoring is warranted since prior studies have demonstrated a high penetrance by 60 years of age. Early manifestation of disease can be subtle and so specific testing for B and NK cell by flow cytometry may be warranted (16, 17).

The clinical presentation of our patient adds to the wide range of clinical manifestations of GATA2 deficiency, and suggests how a stepwise, multidisciplinary diagnostic approach can aid in timely diagnosis and management for infants with severe T-cell lymphopenia. There is much more to learn about GATA2 deficiency and its interaction with additional genetic or environmental factors, that predisposes them to severe complications even after curative therapies (10, 18–20).

## Conclusion

The presence of undetectable TRECs is an excellent method to identify patient with inborn errors of immunity beyond SCID and genetic testing, tailored to the center clinical expertise level, should follow negative molecular testing for SCID to provide prompt diagnosis and management of these patients.

## Data Availability

The original contributions presented in the study are included in the article/Supplementary Material, further inquiries can be directed to the corresponding author/s.
